# Escore de Cálcio Coronariano. Existe Diferença entre os Subtipos de Acidente Vascular Cerebral Isquêmico?

**DOI:** 10.36660/abc.20201192

**Published:** 2020-12-01

**Authors:** Millene Rodrigues Camilo, Octávio Marques Pontes-Neto

**Affiliations:** Departamento de Neurociências e Ciências do Comportamento Faculdade de Medicina de Ribeirão Preto Universidade de São Paulo Ribeirão PretoSP Brasil Divisão de Neurologia - Departamento de Neurociências e Ciências do Comportamento - Faculdade de Medicina de Ribeirão Preto - Universidade de São Paulo, Ribeirão Preto, SP - Brasil

**Keywords:** Acidente Vascular Cerebral, Doença da Artéria Coronária, Escore de Cálcio, Isquemia Miocárdica, Aterosclerose

A doença isquêmica cardíaca é uma importante causa de morte em pacientes com acidente vascular cerebral (AVC) durante um seguimento de longo prazo.^1 , 2^ Os sobreviventes de AVC isquêmico têm uma alta prevalência de doença arterial coronariana (DAC) assintomática.^3^ Na verdade, metade dos pacientes sem histórico cardíaco têm algum grau de placa aterosclerótica coronariana e um terço apresenta mais de 50% de estenose.^4^ Para avaliar o risco de DAC, um escore de estratificação não invasivo baseado na extensão e densidade do cálcio arterial coronariano (CAC) por tomografia computadorizada foi proposto por Agatston et al.^5^ A medida do CAC para melhorar a previsão do risco clínico para eventos cardiovasculares em adultos assintomáticos selecionados é a recomendação de diretrizes mundiais.^6 - 10^ Embora haja uma forte associação entre a aterosclerose e DAC subclínica, isso ainda é incerto para pacientes com AVC não-aterosclerótico.^1 , 11^

Nesta edição da ABC Cardiol, Negrão et al.,^12^ conduziram um estudo transversal para comparar o escore de cálcio das artérias coronárias (ECAC) entre pacientes com AVC isquêmico aterosclerótico e não-aterosclerótico que foram admitidos no Hospital de Reabilitação. Dos 244 pacientes avaliados, 80 (33%) foram incluídos no grupo de etiologia aterosclerótica. O grupo não-aterosclerótico foi representado pelas demais etiologias, como cardioembolismo (30%), oclusão de pequenas artérias (15%), outras causas (6%) e causa indeterminada (16%). Embora não tenha havido diferença no risco de DAC entre os dois grupos, a idade ≥60 anos foi um preditor independente para alto risco de DAC (OR 3,52; IC95% 1,72-7,18).^12^

Este estudo forneceu percepções relevantes que devem ser abordadas. Em primeiro lugar, AVC e DAC têm uma relação estreita, compartilhando fatores de risco comuns.^1 , 3^ Mesmo entre pacientes jovens com AVC, a prevalência desses riscos é substancial. Um estudo publicado recentemente relatou que os três fatores de risco mais comuns para AVC em jovens eram hipertensão arterial, distúrbios lipídicos e fatores relacionados ao estilo de vida. Mais da metade dos pacientes apresentou pelo menos dois fatores de risco independentes para AVC.^13^

Da mesma forma, a população com AVC no presente estudo era relativamente jovem (58,4 ± 6,8 anos), mas apresentava uma frequência elevada de fatores de risco. Em segundo lugar, a síndrome coronariana aguda resulta principalmente de aterosclerose de grandes vasos, enquanto os pacientes com AVC isquêmico constituem um grupo heterogêneo, incluindo cinco categorias de classificação etiológica (aterosclerose de grandes artérias; cardioembolismo; oclusão de pequenas artérias; outra causa determinada; e causa indeterminada).^14^ Além disso, é bem conhecido que há variação no risco de DAC de acordo com o mecanismo do AVC. Pacientes com dissecção arterial, outras arteriopatias não-ateroscleróticas e embolia paradoxal parecem ter baixo risco de DAC. Enquanto aqueles com AVC cardioembólico, atribuído principalmente à fibrilação atrial, podem apresentar uma maior probabilidade de eventos coronários. Ao contrário dos extensos dados sobre aterosclerose de artéria extracraniana e DAC, as informações sobre a aterosclerose intracraniana são insuficientes.^11 , 14^ Terceiro, a terapia com estatinas pode ser um fator de confusão na quantificação do ECAC. Como as estatinas podem reduzir as placas fibrolipídicas e promover microcalcificação, também pode levar ao aumento do ECAC.^15^ Por fim, como os autores indicaram, houve um possível viés de seleção, excluindo pacientes com limitação bastante grave ou baixa demanda de recuperação. Portanto, esses resultados devem ser interpretados levando-se em consideração essas limitações e as características da coorte, antes de considerar-se uma ampla generalização.

No geral, não foi uma surpresa que o ECAC tenha sido incapaz de diferenciar a etiologia do AVC. O mais interessante é que os pacientes com AVC aterosclerótico e não-aterosclerótico apresentaram proporções semelhantes de risco de DAC. Grandes estudos com seguimento mais longo devem ser realizados para determinar o valor do ECAC para estratificação individual de risco de DAC em pacientes com AVC isquêmico, independentemente da etiologia. As ferramentas de predição de risco são críticas para estabelecer estratégias de intervenção, com o objetivo de prevenir eventos coronarianos maiores em pacientes com AVC.
