# Supplementary material for: Escore de Cálcio Coronariano. Existe Diferença entre os Subtipos de Acidente Vascular Cerebral Isquêmico?
Source: Arq Bras Cardiol. 2020 Dec 1;115(6):1152–3. [Article in Portuguese] doi: 10.36660/abc.20201192 (PMC8133728; doi:10.36660/abc.20201192)
Supplement: Translation

# Translation: Coronary Calcium Score. Is There a Difference among Ischemic Stroke Subtypes?

**Authors:** Millene Rodrigues Camilo, Octavio Marques Pontes-Neto

**Affiliations:** Brazil Divisão de Neurologia - Departamento de Neurociências e Ciências do Comportamento - Faculdade de Medicina de Ribeirão Preto - Universidade de São Paulo, Ribeirão Preto, SP - Brazil

**Keywords:** Stroke, Coronary Artery Disease, Calcium Score, Myocardial Ischemia, Atherosclerosis

Ischemic heart disease is an important cause of death in stroke patients during a long-term follow-up.^1 , 2^Ischemic stroke survivors have a high prevalence of asymptomatic coronary artery disease (CAD).^3^Indeed, half of those with no cardiac history have some degree of coronary atherosclerotic plaques and one-third have more than 50% of coronary stenosis.^4^For the assessment of CAD risk, a non-invasive stratification score based on the extent and density of computed tomography coronary artery calcium (CAC) was proposed by Agatston et al.^5^The measurement of CAC to improve clinical risk prediction for cardiovascular events in selected asymptomatic adults is the recommendation of worldwide guidelines.^6 - 10^Although there is a strong association between atherosclerosis and subclinical CAD, it is remains uncertain for non-atherosclerotic stroke patients.^1 , 11^

In this issue of ABC Cardiol, Negrão et al.^12^conducted a cross-sectional study to compare the coronary calcium score (CCS) between atherosclerotic and non-atherosclerotic ischemic stroke patients who were admitted at Rehabilitation Hospital. Of 244 evaluated patients, 80 (33%) were included in the atherosclerotic etiology group. The non-atherosclerotic group was represented by the remaining etiologies, such as cardioembolism (30%), small artery occlusion (15%), other causes (6%), and undetermined cause (16%). Although there was no difference in CAD risk between those groups, age ≥60 years was an independent predictor for high CAD risk (OR 3.52; 95%CI 1.72-7.18).^12^

This study provided relevant insights that should be addressed. First, stroke and CAD have a close association, sharing common risk factors.^1 , 3^Even among young stroke patients, the prevalence of these risks is substantial. A recent published study reported that the three most common risk factors for stroke at a young age were arterial hypertension, lipid disorders and lifestyle-related factors. More than half of the patients had at least two independent risk factors for stroke.^13^

Likewise, the stroke population in the present study showed a relatively young age (58.4 ± 6.8 years), but had a high frequency of risk factors. Second, acute coronary syndrome results mainly from large-vessel atherosclerosis, whereas ischemic stroke patients are a heterogeneous group, including five categories of etiology classification (large-artery atherosclerosis; cardioembolism; small artery occlusion; other determined cause; and undetermined cause).^14^In addition, it is well recognized that there is a variation in CAD risk according to the stroke mechanism. Patients with artery dissection, other nonatherosclerotic arteriopathies, and paradoxical embolism seem to be at low CAD risk. While those with cardioembolic stroke, mainly attributed to atrial fibrillation, may have a higher likelihood of coronary events. As opposed to a large amount of data on extracranial artery atherosclerosis and CAD, insufficient information is available about intracranial atherosclerosis.^11 , 14^Third, statin therapy may be a confounder in CCS quantification. Since statins can reduce the fibrolipid plaques and promote micro-calcification, it might also lead to an increase in CCS.^15^Finally, as the authors pointed out, there was a possible selection bias, excluding either patients with quite severe limitation or low recovery demand. Therefore, these results should be interpreted taking into account these limitations and the cohort characteristic, before jumping to broad generalization.

Overall, it was not a surprise that the CCS itself was unable to distinguish stroke etiology. Most interestingly, atherosclerotic and non-atherosclerotic stroke patients showed similar proportions for CAD risk. Large studies with longer follow-up periods should be undertaken to determine the CCS value for individual CAD risk stratification in ischemic stroke patients regardless of the etiology. Risk prediction tools are critical for intervention strategies, aiming to prevent major coronary events in stroke patients.
